# Alternative to improve palliative care for all patients and families in critical care units: development and preliminary evaluation following MRC guidance of the King's Psychosocial, Assessment and Care tool

**DOI:** 10.1186/cc12466

**Published:** 2013-03-19

**Authors:** I Higginson, C Rumble, J Koffman, P Hopkins, S Heenen, W Prentice, R Burman, S Leonard, O Dampier, J Noble, M Morgan, C Shipman

**Affiliations:** 1 King's Health Partners AHSC, London, UK

## Introduction

More than one in five people admitted to an ICU will die there. Research has highlighted concerns about support for patients and families and decision-making in this context [1,2]. Here, we describe the development and evaluation of a tool to improve palliative care in a 32-bed general ICU in a central London teaching hospital.

## Methods

Medical Research Council guidance for complex interventions Phase 0 to I comprised literature review, theoretical modelling, observation and qualitative interviews and focus groups with staffand families exploring concerns and views of interventions identified in the literature review. Phase II comprised intervention development, implementation and evaluation of tool feasibility and effects using staff survey, observation, audit of records and relative survey.

## Results

Phase I: 47 staffand 24 family members were interviewed. The short time between decisions for treatment withdrawal and death, plus concerns for support management, communication and decision-making, highlighted a need to ensure excellent psychosocial assessment for all. Phase II: as part of integrated care guidelines, we developed the King's Psychosocial Assessment and Care tool (K-PACE). K-PACE is used for all patients entering the ICU, completed within 24 hours of admission. It contains psychosocial assessment of the family and patient needs, and identifies key individuals for contact. Educational training was supported by K-PACE and was implemented in two waves. Post-implementation survey of 95 ICU stafffound that most (80%) were aware of K-PACE. Eighty-two per cent of nurses but only 17% of doctors had completed the tool. In total, 158/213 (74%) family members responded to the survey (additionally three patients responded). There were high levels of satisfaction for symptom control and psychosocial care but concerns continued regarding explanation of treatment and care.

## Conclusion

K-PACE is a feasible tool to improve the palliative care of patients and their families in the ICU. Further refinement is needed and planned, with consideration of roll-out into the wider medical centre.
